# Effect of a Multistarter Yeast Inoculum on Ethanol Reduction and Population Dynamics in Wine Fermentation

**DOI:** 10.3390/foods10030623

**Published:** 2021-03-15

**Authors:** Xiaolin Zhu, María-Jesús Torija, Albert Mas, Gemma Beltran, Yurena Navarro

**Affiliations:** Department of Biochemistry and Biotechnology, Faculty of Oenology, University Rovira i Virgili (URV), Marcel·lí Domingo 1, 43007 Tarragona, Catalonia, Spain; xiaolin.zhu@estudiants.urv.cat (X.Z.); mjesus.torija@urv.cat (M.-J.T.); albert.mas@urv.cat (A.M.); yurenadelosangeles.navarro@urv.cat (Y.N.)

**Keywords:** wine, PMA-qPCR, *Metschnikowia pulcherrima*, *Torulaspora delbrueckii*, *Zygosaccharomyces bailii*, mixed fermentation, coinoculation, sequential fermentation

## Abstract

Microbiological strategies are currently being considered as methods for reducing the ethanol content of wine. Fermentations started with a multistarter of three non-*Saccharomyces* yeasts (*Metschnikowia pulcherrima* (Mp), *Torulaspora delbrueckii* (Td) and *Zygosaccharomyces bailii* (Zb)) at different inoculum concentrations. *S. cerevisiae* (Sc) was inoculated into fermentations at 0 h (coinoculation), 48 h or 72 h (sequential fermentations). The microbial populations were analyzed by a culture-dependent approach (Wallerstein Laboratory Nutrient (WLN) culture medium) and a culture-independent method (PMA-qPCR). The results showed that among these three non-*Saccharomyces* yeasts, Td became the dominant non-*Saccharomyces* yeast in all fermentations, and Mp was the minority yeast. Sc was able to grow in all fermentations where it was involved, being the dominant yeast at the end of fermentation. We obtained a significant ethanol reduction of 0.48 to 0.77% (*v*/*v*) in sequential fermentations, with increased concentrations of lactic and acetic acids. The highest reduction was achieved when the inoculum concentration of non-*Saccharomyces* yeast was 10 times higher (10^7^ cells/mL) than that of *S. cerevisiae*. However, this reduction was lower than that obtained when these strains were used as single non-*Saccharomyces* species in the starter, indicating that interactions between them affected their performance. Therefore, more combinations of yeast species should be tested to achieve greater ethanol reductions.

## 1. Introduction

In recent years, the average ethanol concentration in wine has increased, mainly due to climate change and consumer preference for wine styles [1,2,3]. Different strategies have been applied to reduce ethanol production in wine, such as decreasing the leaf area to lower the sugar content in grape berries, reducing the maturity of grapes, removing sugar from the grape must, developing or screening low-alcohol wine yeasts and removing alcohol from wine (reviewed by [4]). Compared with microbiological strategies, other strategies might have negative effects on wine, such as delaying maturity, reducing the yield of berries, causing a significant reduction in anthocyanins, soluble solids and volatile compounds, and decreasing the wine color [5,6,7,8]. Therefore, microbiological strategies were considered to be effective and accompanied by smaller impacts on the wine sensory profile and quality. In particular, the use of non-*Saccharomyces* yeast strains for reducing the alcohol content of wines has been proven to improve the wine aroma complexity and has become a consistent proposal [9,10,11].

Non-*Saccharomyces* yeasts have been applied in fermentations to reduce ethanol using different inoculation strategies. For example, several studies have shown that single-culture fermentations with *Hanseniaspora uvarum*, *Lachancea thermotolerans*, *Metschnikowia pulcherrima*, *Starmerella bombicola*, *Starmerella bacillaris, Zygosaccharomyces bailii*, *Zygosaccharomyces bisporus*, and *Zygosaccharomyces sapae* species are able to reduce the ethanol content in wine [10,12,13,14,15,16,17,18,19]. However, most of these single yeast fermentations became stuck. To solve this problem, mixed fermentations with non-*Saccharomyces* yeasts and *Saccharomyces cerevisiae* have been proposed, demonstrating that simultaneous inoculation of *S. cerevisiae* with *L. thermotolerans, S. bacillaris* or *M. pulcherrima* species produced a reduction in ethanol [10,12,14,15,20]. In addition, to achieve this purpose, most researchers preferred to use non-*Saccharomyces* yeasts (*Hanseniaspora osmophila*, *H. uvarum*, *L. thermotolerans*, *M. pulcherrima*, *S. bombicola*, *Torulaspora delbrueckii* and *Z. bailii* species) in sequential fermentations, with *S. cerevisiae* inoculated at 48 h, 72 h, or when 50% of the sugar from grape must was consumed [11,12,17,21,22,23,24].

Due to the growing interest in mixed fermentations, it is necessary to understand the behavior and interactions of strains throughout the fermentation process. Thus, the population dynamics of yeasts have become a key factor in the study of yeast interactions. The traditional microbial counting method usually uses different solid media, such as Wallerstein Laboratory Nutrient (WLN) and lysine agar, to distinguish or isolate different yeast species based on their dissimilar morphological characteristics or selective growth [25,26]. However, this is not an effective method of discrimination when two or more non-*Saccharomyces* yeast species are simultaneously inoculated in mixed fermentations because some of them show similar morphological profiles. In addition, for an accurate evaluation of the population dynamics of different yeast species, we have to consider the existence of viable but nonculturable (VBNC) cells caused by the different metabolic statuses of cells. In these cases, the use of the plating method would not be appropriate because those VBNC cells would not be detectable [27].

Compared with the classical method, researchers have confirmed that quantitative real-time polymerase chain reaction (qPCR) is a more sensitive and specific technique for the detection of nucleic acids, and it shows a wide detection range for different cell concentrations, usually from 10 to 10^8^ cells/mL [28,29,30]. This sensitive and low-detection limit method provides the possibility to detect species present in low quantities in the mixed fermentation process. However, the population of yeasts is often overestimated from qPCR because the method does not discriminate the DNA of living and dead cells in fermenting must [31,32]. To distinguish the population of living cells from that of dead cells, DNA-binding dyes, such as ethidium monoazide (EMA) and propidium monoazide (PMA), are applied to must samples as a pretreatment before DNA extraction. The qPCR analysis of these samples quantifies only living cells because the living cell membrane is impermeable to DNA-binding dye, and therefore, it will only bind to free DNA from dead cells, avoiding its amplification. Nocker et al. [33] showed that PMA is more effective than EMA, which is the reason why PMA has been favored by researchers in the counting of living cells in recent years [34,35,36,37,38].

Most articles have focused on evaluating the effects of single strains or mixed starters composed of one *S. cerevisiae* strain and one non-*Saccharomyces* species. However, this work aims to test a multistarter culture consisting of several non-*Saccharomyces* species previously selected for their ability to reduce ethanol [39] and a commercial *S. cerevisiae* strain. The use of multiple selected species as the inoculum may improve ethanol reduction, as well as the overall complexity of wines [32].

The aim of the present work was to analyze the effect of the mixed inoculation of *S. cerevisiae* and several non-*Saccharomyces* yeasts on ethanol reduction and population dynamics. Three non-*Saccharomyces* strains belonging to *M. pulcherrima*, *T. delbrueckii* and *Z. bailii* species, which have been demonstrated to have the ability to reduce ethanol in single fermentations [39], were simultaneously inoculated into the must. To determine the best inoculation protocol to achieve ethanol reduction, different mixed inoculation conditions with *S. cerevisiae* were evaluated (sequential or coinoculation at different inoculation ratios). The population dynamics of the different yeasts were assessed by WLN counting and PMA-qPCR analysis during the fermentation process, and the concentrations of the main organic compounds were analyzed by HPLC in the resulting wines.

## 2. Materials and Methods

### 2.1. Yeast Strains

Four different yeast strains were used in this study: the commercial wine yeast *S. cerevisiae* (Lalvin QA23^®^, referred to as Sc) from Lallemand Inc. (Montreal, QC, Canada), *M. pulcherrima* 51 (Mp) and *T. delbrueckii* Priorat (Td), selected from the Priorat Appellation of Origin (URV collection, Tarragona, Spain) [9], and *Z. bailii* CECT 11043 (Zb), obtained from the Spanish Type Culture Collection. These strains were previously selected for their ability to produce low ethanol yield [39] when several strains of each species were tested.

The strains were stored at −80 °C in YPD liquid medium (2% (*w*/*v*) glucose, 2% (*w*/*v*) yeast extract, and 1% (*w*/*v*) peptone, Cultimed, Barcelona, Spain) with 40% (*v*/*v*) glycerol. Before starting the fermentations, the yeasts were grown at 28 °C in YPD agar (YPD liquid with 1.7% (*w*/*v*) agar) and Wallerstein laboratory nutrient (WLN) agar (Becton, Dickinson and Company, Isère, France). The species identification of the four strains was confirmed by PCR-RFLP analysis of 5.8S-ITS rDNA according to Esteve-Zarzoso et al. [40].

### 2.2. Natural Must and Starter Cultures

Natural must (NM) was obtained from Muscat grapes harvested from Finca Experimental Mas dels Frares of Rovira i Virgili University (Constantí, Spain) during the 2019 vintage (after treatment with 50 mg/L SO_2_ and settling, the must had 220 g/L sugars, 4.49 g/L total acidity (as tartaric acid), 77.8 mg/L assimilable nitrogen and a pH of 3.27). The concentration of assimilable nitrogen was determined in a Miura autoanalyzer (EE-MIURAONE Rev., I.S.E. S.r.l., Italy) using an Ammonia and α-Aminic Nitrogen Enzymatic KIT (Tecnología Difusión Ibérica, S.L., Barcelona, Spain) and corrected with diammonium phosphate (Panreac Quimica SA, E.U.) until reaching a final concentration of 250 mg N/L. Before the start of the fermentations, dimethyl dicarbonate (0.2 mL/L) (ChemCruz, Santa Cruz Biotechnology, America) was added to NM and kept at 4 °C for 24 h to eliminate endogenous microorganisms. The absence of microorganisms after this treatment was confirmed by plating must samples with no dilution and 10-fold dilution on YPD agar.

The starter cultures were performed by transferring a single colony from YPD agar to YPD liquid medium and incubating it for 24 h (*S. cerevisiae*) or 48 h (non-*Saccharomyces*) at 28 °C with a stirring rate of 120 rpm in an orbital shaker. After incubation, the cells were counted in a Neubauer chamber (Leica Microsystems GMS QmbH, Leica, Germany) and inoculated at the indicated concentrations into the NM.

### 2.3. Fermentation Trials in Natural Must

Eight different mixed fermentations were carried out in this study (Table 1). Initially, all fermentations were simultaneously inoculated with three non-*Saccharomyces* strains at two different concentrations: 10^6^ cells/mL (fermentations named 1CN) or 10^7^ cells/mL (fermentations named 10CN). Additionally, Sc was inoculated into these two mixed non-*Saccharomyces* starters at the same moment (coinoculated fermentations were named 1CA and 10CA) and at 48 or 72 h later (sequential fermentations named 1S48, 1S72, 10S48 and 10S72, respectively), always at the same concentration (10^6^ cells/mL). A single fermentation with Sc (10^6^ cells/mL) was used as the control fermentation (C).

Triplicate fermentations were conducted in 250 mL glass bottles with 230 mL of NM and incubated at 22 °C with stirring at 120 rpm. The bottle cap had two ports—one connected to a 0.22 μm filter (Dominique Dutscher, Brumath, France) for gas flow and the other clamped by an iron clip for sampling. Before inoculation with Sc in sequential fermentations, the concentration of assimilable nitrogen in the fermented must was determined and supplemented, if needed, to 100 mg/L. Fermentations were monitored by measuring the must density with an electronic densitometer (Densito 30PX Portable Density Meter, Mettler Toledo, Hospitalet de Llobregat, Spain). Fermentations were considered finished when the residual sugars were below 2 g/L, checked by D-glucose/D-fructose enzymatic assays (Biosystems S.A., Barcelona, Spain) in the Miura autoanalyzer, or arrested when the must density did not decrease for more than two days. Samples from the end of fermentation were centrifuged at 7800 rpm for 5 min, and supernatants were frozen at 20 °C until chemical analysis.

### 2.4. Colony Counting

The yeast population during fermentation was analyzed by colony growth on WLN plates according to the different colony morphologies of *Saccharomyces* and non-*Saccharomyces* strains [35]. Briefly, samples were serially diluted in sterilized Milli-Q water from a Milli-Q purification system (Millipore S.A.S., Molsheim, France). The number of colony-forming units per milliliter (CFU/mL) was determined by plating 100 μL of three appropriately chosen dilutions on WLN agar. The plates were incubated at 28 °C for 2 or 3 days.

### 2.5. PMAxx Treatment

To obtain only the DNA of living cells, PMAxx^TM^ viability dye (Biotium Inc., Fremont, CA, USA) was added to must samples as previously described by Navarro et al. [38]. Briefly, selected samples of must (1 mL) were centrifuged, and the pellets were washed with sterilized distilled water and treated with 25 μM PMAxx. After incubation for 10 min in darkness, PMAxx was permanently linked to the DNA of dead cells by subjecting samples twice to light for 30 s, with an interval of 1 min on ice. Pellets were recovered by centrifugation and frozen until DNA extraction.

### 2.6. DNA Extraction and qPCR Analysis

DNA was extracted using the DNeasy Plant Mini Kit (QIAGEN GmbH, Hilden, Germany) following the instructions of the manufacturer. Cell quantification of the different yeast species was conducted by species-specific qPCR using the primers CESP-F/SCER-R for *S. cerevisiae* [31], MP2-F/MP2-R for *M. pulcherrima* [41], Tods L2/Tods R2 for *T. delbrueckii* [42] and ZBF1/ZBR1 for *Z. bailii* [29] (all primer sequences are shown in Table A1). For all samples, qPCR was performed in triplicate using TB Green^TM^ Premix Ex Taq^TM^ II (2×) (Takara Bio Inc., Kusatsu, Japan) with a final volume of 20 μL (2 μL of DNA, 0.8 μL of each primer, 0.08 μL of ROX Reference Dye (50×), 10 μL TB Green Premix Ex Taq II (2×) and 6.32 μL of sterilized Milli-Q water) on a QuantStudio^TM^ 5 real-time PCR instrument (Applied Biosystems by Thermo Fisher Scientific, Waltham, MA, USA). A 96-well nonskirted PCR plate (4titude^®^ Ltd., Wotton, UK) was used for the reaction. The amplification reaction was one cycle of 95 °C for 1 min and 40 cycles of 95 °C for 5 s and 60 °C for 35 s, followed by a dissociation step. Milli-Q water was used as a negative control. Ct (the cycle threshold) was determined using Thermo Fisher Scientific software (Waltham, MA, USA).

Standard curves were calculated for each species with and without PMAxx treatment, as described by Navarro et al. [38] but slightly modified. After incubating the yeast colony in YPD liquid medium at 28 °C for 24 h, samples with 10^8^ cells/mL were collected in triplicate and centrifuged at 10,000 rpm for 2 min. The pellet was washed once with 1 mL sterilized distilled water and was subjected to PMAxx treatment as previously described. The pellet was stored at −20 °C until DNA extraction. Standard curves were created by plotting the average Ct values of a tenfold serial dilution of DNA from 10^8^ to 10 cells/mL against the log of cells/mL.

### 2.7. Chemical Analysis

Residual sugars of samples at the end of fermentation were quantified by D-glucose/D-fructose enzymatic assays. Ethanol, glycerol and organic acids (citric acid, malic acid, tartaric acid, acetic acid, lactic acid and succinic acid) in the samples were determined by high-performance liquid chromatography (HPLC) using an Agilent 1100 (Agilent Technologies, Waldbronn, Germany) as previously described by Quirós et al. [43] and Zhu et al. [39].

### 2.8. Statistical Analysis

All graphs were generated using GraphPad Prism^®^ version 8 (GraphPad Software, San Diego, CA, USA). The results are expressed as the mean ± standard deviation (SD). Statistically significant differences (one-way ANOVA) were analyzed by IBM SPSS Statistics version 23.0 (IBM, NY, USA). The ethanol yield was calculated with the Equation (1).

Ethanol yield (g/g) = ethanol production (g/L)/sugar consumption (g/L)(1)

## 3. Results

### 3.1. Fermentation Kinetics

**Figure 1:**
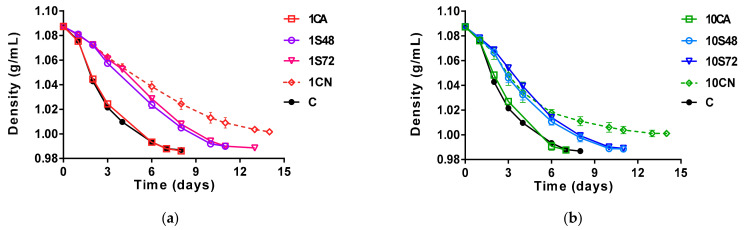
Density of single and mixed fermentations of *S. cerevisiae* and non-*Saccharomyces* yeasts with different inoculation conditions (see Table 1). The initial non-*Saccharomyces* inoculum size was 10^6^ cells/mL (**a**) and 10^7^ cells/mL (**b**).

The fermentation kinetic profiles obtained under the different inoculation conditions, measured as must density reduction, are shown in Figure 1. The fastest fermentations were those with single inoculation with Sc and with the coinoculation of all the strains at the same time (1CA and 10CA), which showed similar fermentation profiles and completed fermentations in 7–8 days (10CA completed the fermentation 1 day earlier than that of Sc alone (C)). The fermentation kinetics were less affected by the inoculum concentration of non-*Saccharomyces* yeasts but were influenced by the inoculation time of Sc. The sequential inoculations, in which only non-*Saccharomyces* strains were fermented for 48–72 h, resulted in slower fermentations, finishing all the sugars in 11 (1S48, 10S48, 10S72) or 13 days (1S72) (Figure 1). These fermentations were slightly slower when Sc was inoculated later, at 72 h, mainly in the lower non-*Saccharomyces* inoculum (1S72). Finally, fermentations with only non-*Saccharomyces* species (1CN and 10CN) showed the slowest fermentation kinetics, getting stuck on the 13th day, at densities of 1.002 and 1.001 g/mL, respectively (Figure 1a,b). In general, the fermentations with non-*Saccharomyces* strains inoculated at 10^7^ cells/mL showed slightly faster fermentation kinetics than did those inoculated at 10^6^ cells/mL (Figure 1a,b).

### 3.2. Yeast Population Dynamics

**Figure 2:**
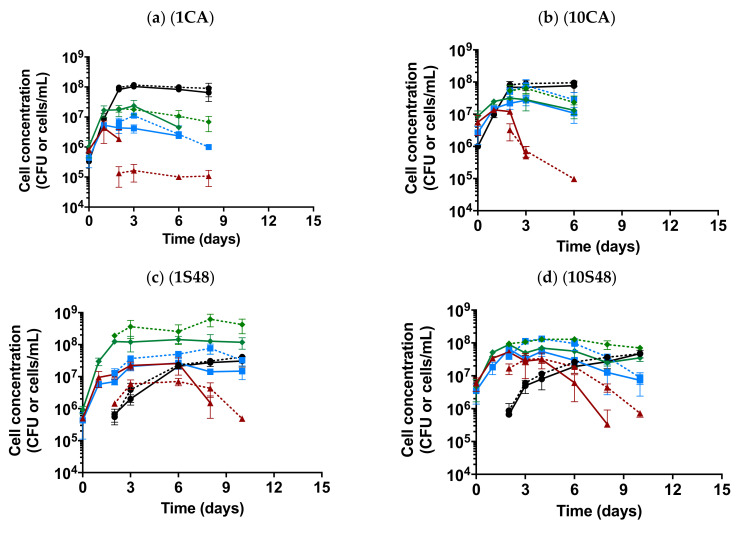

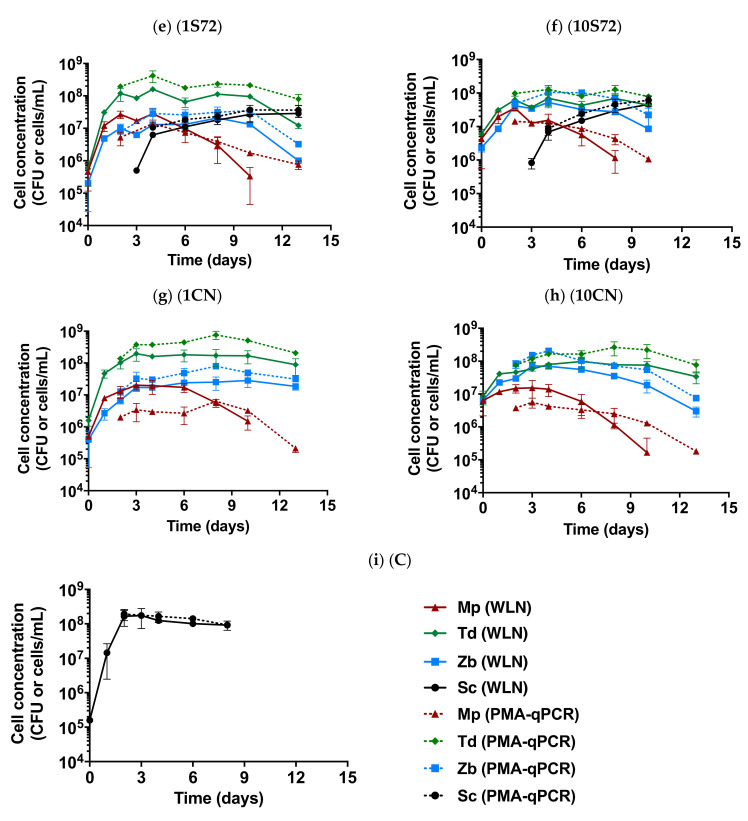
Yeast population analysis based on Wallerstein Laboratory Nutrient (WLN) plates (solid lines 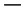) and qPCR with PMAxx treatment (dotted lines 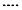), determined in the different fermentative conditions: non-*Saccharomyces* multistarter coinoculated with (**a**,**b**) and without (**g**,**h**) *S. cerevisaie (Sc)*; non-*Saccharomyces* multistarter with sequential inoculation of Sc at 48 h (**c**,**d**) or 72 h (**e**,**f**) and the single Sc fermentations (**i**). The initial non-*Saccharomyces* inoculum size was 10^6^ cells/mL (**a**,**c**,**e**,**g**) and 10^7^ cells/mL (**b**,**d**,**f**,**h**). Each species is shown in different colors (Mp in red, Td in green, Zb in blue and Sc in black).

To study the yeast population dynamics during single or mixed fermentations, the growth of different species was obtained by different methodologies (Figure 2). On one hand, viability was determined during the fermentation process by plating the samples on WLN agar. Due to the different colony morphologies of the four yeast species on WLN agar [35], the colonies of these four strains could be counted on the same plate. On the other hand, PMA-qPCR analysis was performed to quantify the living yeast population using specific primers for each species. We applied PMA-qPCR from the second day of fermentation because the adaptation of the cells to the medium may produce an underestimation of the population by qPCR [38].

First, the standard curves were calculated by plotting the Ct values (<30) of DNA from 10^3^ to 10^8^ cells/mL (*M. pulcherrima* was from 10^4^ to 10^8^ cells/mL) against the log input cells/mL (Table A2), with efficiencies between 87.92% and 98.83%.

After yeast inoculation, all strains were able to grow in fermentation media and showed different population dynamics, as seen in the results obtained from WLN plates. In fermentations in which each non-*Saccharomyces* species was inoculated at 10^6^ cells/mL, the population of non-*Saccharomyces* yeasts grew rapidly on the first day (Figure 2a,c,e,g). Td quickly increased the population, reaching up to 2 × 10^8^ CFU/mL on the third day. The Td population was then stable until the end of fermentation, becoming the dominant yeast in all mixed inoculations, except in the coinoculated fermentations with Sc (1CA and 10CA, Figure 2a,b), in which Sc significantly impaired growth, with the fermentation reaching a maximum Td population of only 10^7^ CFU/mL under 1CA conditions. Mp reached a maximum population of 2.7 × 10^7^ CFU/mL in the sequential or non-*Saccharomyces* coinoculations, with densities higher than that of Zb within the first days but decreasing after the sixth day under most conditions (the Mp decrease occurred much earlier in the coinoculation with Sc, 1CA). In the case of Zb, the maximum growth was reached in the late fermentation stage, with a population of 2.8 × 10^7^ CFU/mL, and was maintained rather stably until the end of fermentation.

The population dynamics in fermentations inoculated with 10^7^ cells/mL of each non-*Saccharomyces* yeast differed from those inoculated at 10^6^ cells/mL (Figure 2b,d,f,h). In those fermentations, Td and Zb presented similar population dynamics, and although Td was again the yeast with the highest population among all non-*Saccharomyces* yeasts, the difference relative to Zb was not as large (Td reached 9.7 × 10^7^ CFU/mL and Zb 7.0 × 10^7^ CFU/mL). Mp achieved the highest population (5.0 × 10^7^ CFU/mL) on the second day and became the minority strain until the end of fermentation.

Regarding the *S. cerevisiae* dynamics, the dominance was only achieved in both coinoculated fermentations (1CA and 10CA), reaching a similar concentration to that of the Sc single inoculation on the third day of fermentation (1.8 × 10^8^ CFU/mL, Figure 2a,b). However, the growth of Sc was restricted in sequential fermentations, where its population increased gradually until the end of fermentation, achieving up to 4.7 × 10^7^ CFU/mL (Figure 2c–f). In these sequential fermentations, Sc together with Td were the majority strains at the end of the fermentation. This loss of the full imposition was not due to the lack of assimilable nitrogen, since supplementation to 100 mg/L was carried out in the deficient fermentations (1S72, 10S48 and 10S72) before the inoculation of Sc (as detailed in Table A3).

As already mentioned, during the fermentation process, Td and Zb colonies were observed until the end of fermentation (except in fermentation 1CA, where Td and Zb were absent at the end point), while Mp was absent on WLN agar after mid-fermentation. However, PMA-qPCR analysis allowed the detection of Mp until the end of fermentation, with the population ranging from 1 × 10^5^ to 1.1 × 10^6^ cells/mL, as well as that of Td and Zb at the end of fermentation in 1CA (Figure 2a). After the second day of fermentation, the trends of the yeast populations of Td, Zb and Sc obtained by PMA-qPCR analysis were similar to those of the WLN counting, although the cell concentrations obtained by PMA-qPCR were higher, reaching a difference of one order of magnitude for most non-*Saccharomyces* yeasts (Figure 2). Specifically, for the Td, Zb and Sc strains, the maximum populations obtained by PMA-qPCR from mixed fermentations reached 7.6 × 10^8^, 1.7 × 10^8^ and 1.2 × 10^8^ cells/mL, respectively. In contrast, the population of Mp obtained by PMA-qPCR analysis before mid-fermentations was lower than that obtained by WLN counting in the first 6 days for fermentations inoculated with 10^6^ cells/mL and in the first 3 days for fermentations inoculated with 10^7^ cells/mL.

### 3.3. Main Fermentation Byproducts

To detect the ethanol reduction of the different inoculated fermentations and the metabolic characteristics of yeasts under the different conditions, the residual sugar, ethanol production and main byproducts at the end of fermentation were analyzed, as shown in Table 2.

**Figure 3:**
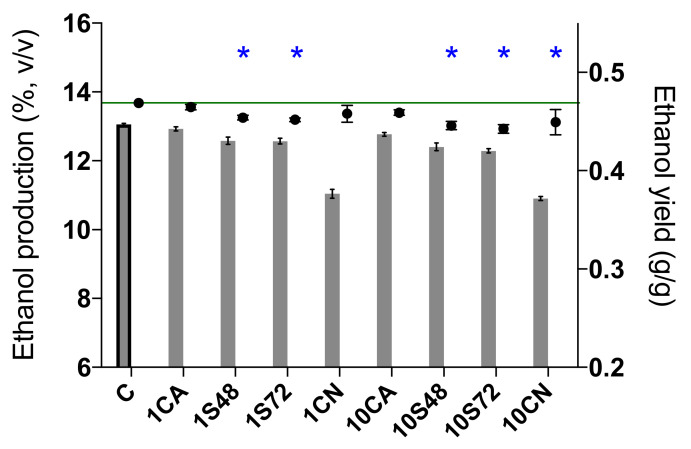
Ethanol production (%, *v*/*v*) and ethanol yield (g/g) at the end of single and mixed fermentations. * indicates statistically significant differences from the control sample (C) (LSD (least significant difference) test, *p* < 0.05). The value of the green line is 0.47 g/g (ethanol yield of C). 1CN and 10CN were stuck fermentations.

All fermentations with a mixed inoculum of non-*Saccharomyces* and Sc were completed, with less than 2 g/L residual sugars. However, the fermentations with only non-*Saccharomyces* strains, 1CN and 10CN, presented higher concentrations of residual sugars due to the stagnation of these two conditions (Table 2). Among all fermentations, the control fermentation (C) had the highest ethanol production (13.06%, *v*/*v*) and ethanol yield (0.47 g/g) (Figure 3). Mixed fermentations decreased ethanol production by 0.13 to 0.77% (*v*/*v*) compared to single fermentation by Sc (C), and this decrease was significant in 5 fermentations (1S48, 1S72, 10CA, 10S48 and 10S72) (Table 2). Fermentation 10S72 showed the highest ethanol reduction with the lowest ethanol yield (0.44 g/g), followed by 10S48, 1S72, 1S48 and 10CA (Table 2, Figure 3). Among these sequential fermentations, our results show a trend in relation to the inoculum concentration of non-*Saccharomyces* yeast, since a higher ethanol reduction was achieved when a higher inoculum of non-*Saccharomyces* was used (0.65 ± 0.11 and 0.77 ± 0.06 with 10^7^ cells/mL vs. 0.48 ± 0.10 and 0.49 ± 0.08 with 10^6^ cells/mL).

Regarding the other fermentation byproducts, the concentration of glycerol in the mixed fermentations was similar to that of single fermentation with Sc (C), except in the 1S48, 1CN and 10CN fermentations, which had lower glycerol contents, although the latter two conditions had residual sugars left. On the other hand, in all fermentations, the concentrations of lactic and acetic acids were higher in the mixed fermentations, although they remained below 0.33 g/L and 0.36 g/L, respectively (Table 2). Surprisingly, more acetic acid was detected at a lower inoculum concentration of non-*Saccharomyces* yeasts (10^6^ cells/mL).

## 4. Discussion

In recent years, non-*Saccharomyces* yeasts have been proposed for use as starters in alcoholic fermentation to reduce the ethanol content in wine [13,15,18,22,39,44,45,46]. Most studies have focused on evaluating the effects of single or mixed starters composed of one *S. cerevisiae* strain and one non-*Saccharomyces* species. Previous studies showed that as single starters, strains of *M. pulcherrima* and *Z. bailii* were not able to complete fermentation or had a low fermentation capacity [21,44], while *T. delbrueckii* has been reported to be a strong fermenter and able to finish fermentations [22,47]. However, fermentations inoculated with several non-*Saccharomyces* species as a multistarter have been poorly studied. In the current study, we evaluated the use of a multistarter of three non-*Saccharomyces* strains in Muscat grape fermentation. These strains were selected in a previous study [39], in which several strains of these three species and other non-*Saccharomyces* species were screened for their ability to reduce ethanol. When this multistarter of non-*Saccharomyces* strains was used, we observed stagnation of fermentation, even though Td was the dominant strain throughout the fermentation process and has shown its ability to complete fermentation when used as a single inoculum [47,48,49]. This lower performance of Td when used in multistarter fermentation could be due to interspecific microbial interactions, such as competition for nutrients [50,51,52], or the production of inhibitory compounds, such as killer toxins or other antimicrobial peptides or vesicles [53,54,55,56], as part of cell-cell interaction mechanisms. The higher population size of the non-*Saccharomyces* inoculum sped the consumption of sugars until mid-fermentation, although this was not enough to complete the fermentation, and similar final populations and residual sugars were obtained under both conditions. Mp was the minority strain under all conditions, and its population decreased significantly throughout the fermentation process, which agrees with previous studies [26,57,58].

As most non-*Saccharomyces* yeasts are incapable of completing alcoholic fermentation, *S. cerevisiae* is usually added, either simultaneously as a coinoculum or sequentially at 24–72 h after non-*Saccharomyces* yeast inoculation. These inoculation strategies reduce the risk of a stuck fermentation [59,60]. Indeed, in the current study, only the fermentations involving *S. cerevisiae* were complete. When all strains were coinoculated, Sc was the dominant strain and became the most abundant yeast at the end of fermentation, regardless of the inoculum concentration of non-*Saccharomyces* yeasts. The dominance of Sc during the fermentation process could be explained by its high ability to tolerate different stresses, such as high ethanol levels, especially relevant in the last stage of fermentation [61]. Additionally, different mechanisms, such as cell-to-cell contact, nutrient competition, secretion of toxic compounds or changes in media [26,62,63,64] could also be responsible of the dominance. The effect of metabolite production and changes in the fermentative medium produced by *S. cerevisiae* has been proven to reduce the competition and persistence of *M. pulcherrima* [65], which agrees with the drastic reduction in the Mp population observed in fermentations when Sc was coinoculated. As described above, the fermentation kinetics were mainly affected by the inoculation time of Sc. Specifically, coinoculated fermentations were faster and finished earlier compared to sequential fermentations, which agrees with previous studies [64,66].

Focusing on competition for nutrients, a key factor in wine fermentation is the availability of assimilable nitrogen. On one hand, in pure-culture fermentations, some non-*Saccharomyces* yeasts (*H. uvarum*, *H. vineae*, *S. bacillaris* and *T. delbrueckii*) consume less assimilable nitrogen than does *S. cerevisiae* [47,67,68]. On the other hand, studies have shown that in sequential fermentations, some non-*Saccharomyces* species, such as *T. delbrueckii*, *L. thermotolerans* and *S. bacillaris*, are capable of consuming almost all assimilable nitrogen within 48 or 72 h before *S. cerevisiae* is inoculated, which could result in incomplete fermentation due to low growth of *S. cerevisiae* [47,69]. In the current study, to avoid stuck fermentations due to nitrogen limitation and to promote the growth of Sc, assimilable nitrogen was restored before Sc inoculation, as non-*Saccharomyces* yeasts had been consuming up to 80% of assimilable nitrogen present in the must. As expected, this addition produced a quick increase in the population of Sc. This conclusion is supported by Medina et al. [68], who also observed an increase in the percentage of the *Saccharomyces* strain after assimilable nitrogen addition.

A critical aspect for studying yeast interactions during fermentation is to evaluate the yeast population, especially living cells. The traditional method used for determining the yeast living cells during alcoholic fermentation is based on colony counting in different culture media, which takes 2 or 3 days and might cause some deviations [27,70]. In mixed or spontaneous fermentations, to differentiate and count the different yeast species, we need to use selective or differential media [27,41]. One of these media is WLN agar, which is able to distinguish different yeast species according to their colony morphology [25,38,71]. In our work, the four yeast species had different colony morphologies on this medium, which allowed their differential counting. In comparison, qPCR analysis is a relatively fast and sensitive technique to simultaneously identify and quantify different targeted yeast species [29,41,72]. However, as described above, this method cannot distinguish the populations of viable and dead cells some DNA-binding dyes, such as PMA, are used and combined with qPCR, allowing the quantification of only the viable cell population [34,38,73].

In the current study, cell counting on WLN plates and PMA-qPCR were used to monitor the viable populations of the different yeast species during the fermentation process. Specific primers for qPCR were available for all species used in the current work [34,38], which presented a high efficiency and good quantification limit. As described above, even though yeast population profiles were similar between WLN counting and PMA-qPCR analysis, they still showed some differences. First, in the coinoculated fermentations with all strains (1CA and 10CA), Sc was the dominant species at the end of fermentation, without detection of these species in the WLN counting in 1CA. Indeed, WLN medium has been described to be useful for quantifying species with similar log populations but is unable to detect species with a population 1 or 2 logs lower than the main species [38], which would explain the inability to detect some species, especially Mp, when its population started decreasing. In contrast, PMA-qPCR sensitively detected the populations of all non-*Saccharomyces* strains until the end of fermentation, even at low concentrations, as observed in previous studies [32,38,41,74]. Surprisingly, the viable cell population of Mp obtained from PMA-qPCR analysis was lower than that from WLN counting before mid-fermentation. This could be explained by the different membrane compositions and permeabilities of this species. Vázquez et al. [75] showed that the cellular lipid composition of *M. pulcherrima* during grape must fermentation contains a high percentage of polyunsaturated fatty acids, which results in more fluid membranes. Moreover, the permeability of the cell membrane is known to be increased upon contact with grape must or ethanol [76]. Thus, this higher permeability could facilitate the penetration of PMAxx into viable Mp cells, resulting in lower quantification by PMA-qPCR analysis. Navarro et al. [38] described this effect in different strains in the early stage of fermentation.

In recent years, *M. pulcherrima*, *T. delbrueckii* and *Z. bailii* have been proven to be species able to reduce ethanol content, mainly in mixed fermentations with *S. cerevisiae*. For example, *M. pulcherrima* was able to reduce 0.7–1.5% (*v*/*v*) ethanol in sequential fermentations in grape must or defined medium [11,15,39,44], while *T. delbrueckii* and *Z. bailii* achieved ethanol reductions of 0.6–1.0% (*v*/*v*) and 0.7–1.8% (*v*/*v*), respectively, in sequential fermentations with *S. cerevisiae* compared to single *S. cerevisiae* fermentation [3,11,39]. All these ethanol reductions were achieved with fermentations initiated by a single non-*Saccharomyces* strain; however, this reduction changed when a multistarter inoculation was used. For example, according to the results from Varela et al. [23], in sequential fermentations with *M. pulcherrima* or *S. uvarum*, the ethanol content decreased by 1.09 and 1.01% (*v*/*v*), respectively, while when those species were simultaneously inoculated as mixed starters with *S. cerevisiae*, the ethanol content decreased by 1.85% (*v*/*v*), showing an additive effect of coinoculation. In the current study, we used a mixed starter of three non-*Saccharomyces* species selected based on their ability to reduce ethanol. Our previous studies showed that the selected Mp, Td and Zb strains were able to reduce the ethanol content by 1.39, 0.84 and 1.02% (*v*/*v*), respectively, in sequential fermentations with Sc (inoculated at 48 h), using the same natural must [39]. In this research, the mixed inoculation of these three species in sequential fermentations with Sc resulted in a lower ethanol reduction (less than 0.77% (*v*/*v*)) compared to sequential fermentation with a single non-*Saccharomyces* strain. Thus, we did not observe an additive effect, as did Contreras et al. [77] or Varela et al. [23]. Instead, those species showed a dissipative impact on reducing ethanol production when used together. Similar results were also observed in the study of Contreras et al. [77], in which sequential fermentations by the single starter of *M. pulcherrima* achieved an ethanol reduction of 1.16–1.76% (*v*/*v*), while sequential fermentations initiated by mixed starters of *M. pulcherrima*, *H. uvarum*, *P. kluyveri* and *T. delbrueckii* reduced the ethanol content by only 0.38% (*v*/*v*). Therefore, Contreras et al. [77] demonstrated that ethanol reduction is very dependent on the yeast combination used in the starter, since a yeast strain with a high ability to reduce ethanol, such as *M. pulcherrima*, was affected by the presence of other yeasts, and as a result, its ability to reduce ethanol could be weakened or strengthened. Moreover, the relative proportion between yeast species during the fermentation could affect the metabolite production. Indeed, comparisons between pure cultures and sequentially inoculated cultures revealed changes in the distribution of carbon fluxes during fermentation [78]. Finally, the initial must composition and nutrient availability can also have an impact in the metabolite production, as previously demonstrated by Seguinot et al. [78].

In addition, our results showed that the inoculum size of non-*Saccharomyces* yeasts also has an impact on ethanol production, with a lower ethanol content in wines inoculated with higher populations of non-*Saccharomyces* yeasts. This agrees with the results obtained by Maturano et al. [79], in which a higher inoculum size of non-*Saccharomyces* yeasts (5 × 10^6^ cells/mL vs. 1 × 10^6^ cells/mL) produced wines with a lower ethanol content. Thus, more combinations of yeast species in terms of both the number and diversity of strains and the amount of each strain inoculated should be tested to find the best combination for ethanol reduction.

Non-*Saccharomyces* yeasts usually differ from *S. cerevisiae* in the distribution of their metabolic flux during fermentation [46,67,78,80]. Indeed, several non-*Saccharomyces* species are able to aerobically respire sugar, which results in altered formation of the main metabolites produced during fermentation, including ethanol, glycerol and organic acids [3,20,45,46]. However, although ethanol production can be decreased when providing non-*Saccharomyces* yeasts with oxygen, this sometimes has undesirable side effects, such as higher acetic acid or ethyl acetate levels [3,81]. In this study, a significant decrease in ethanol levels was observed in mixed fermentations, even without the addition of oxygen and without a detrimental increase in acetic acid. Indeed, higher levels of acetic and lactic acids were obtained in the presence of non-*Saccharomyces* yeast, mainly in sequential inoculations, but the levels were kept below 0.33 g/L for lactic acid and 0.36 g/L for acetic acid, with nondetrimental concentrations for the final wines. On the other hand, the concentration of glycerol did not increase in our reduced-ethanol wines, which implied that the production of glycerol was not the main pathway of ethanol reduction in mixed culture fermentations, as occurred in other studies [39,82]. Nevertheless, the final amounts of acetic and lactic acids are not enough to counterbalance the decrease in ethanol and glycerol. Previous studies had also observed that the reduction of the ethanol yield by some non-*Saccharomyces* strains, could not be fully explained by the overproduction of glycerol or organic acids, suggesting that respiration would be responsible, at least in part, of the poor ethanol yield observed for these strains [83,84]. However, the analysis of the volatile composition of wines (higher alcohols, volatile fatty acids, esters, carbonyl compounds) would help the understanding of the effect of this type of inoculation on the overall complexity of wines [11,85].

In summary, our results confirm that PMA-qPCR analysis is a fast and sensitive method for monitoring the viable cell population dynamics in mixed fermentations of *T. delbrueckii*, *M. pulcherrima*, *Z. bailii* and *S. cerevisiae*. *T. delbruecckii* was the dominant non-*Saccharomyces* species under all conditions, and *M. pulcherrima* was the minority species, being detected by PMA-qPCR throughout fermentation but at lower and decreasing concentrations. The use of a multistarter culture consisting of several non-*Saccharomyces* species previously selected for their ability to reduce ethanol and a *S. cerevisiae* strain resulted in reduced-alcohol wines, even if no aeration was applied. The sequential fermentations obtained ethanol reductions from 0.48–0.77% (*v*/*v*), which were accompanied by increases in the lactic acid and acetic acid contents. Among all fermentations, the highest reduction was obtained in the sequential inoculation with a higher inoculum size (10^7^ cells/mL), when *S. cerevisiae* was added at 72 h. Nevertheless, the ethanol reduction obtained when using a multistarter of non-*Saccharomyces* species was lower than that when using each non-*Saccharomyces* species separately [39], which indicates no additive effect among them but a lower efficiency to deliver this outcome due to microbial interactions. Although not as efficient in reducing ethanol, a multistarter inoculation strategy could have additional benefits, such as improving the aroma profile and overall complexity of wines [23,32]. Nevertheless, the use of several species can also be challenged by winemaking conditions and the initial yeast population; therefore, special care has to be taken in the winery for this kind of procedure to be applied [32,77].

## Figures and Tables

**Table 1:** Fermentation conditions in 230 mL Muscat sterile must. Non-*Saccharomyces* (Mp, Td and Zb) and *S. cerevisiae* (Sc) strains were inoculated at the listed cell concentrations (cells/mL) and times.

Fermentations	Inoculum Procedures	Inoculum Ratios (Mp:Td:Zb:Sc)	Mp	Td	Zb	Sc
1CA	Co-inoculation of all strains	1:1:1:1	1 × 10^6^	1 × 10^6^	1 × 10^6^	1 × 10^6^
1S48	Sequential inoculation of Sc at 48 h	1:1:1:1	1 × 10^6^	1 × 10^6^	1 × 10^6^	1 × 10^6^
1S72	Sequential inoculation of Sc at 72 h	1:1:1:1	1 × 10^6^	1 × 10^6^	1 × 10^6^	1 × 10^6^
1CN	Co-inoculation of non-*Saccharomyces* strains	1:1:1:0	1 × 10^6^	1 × 10^6^	1 × 10^6^	
10CA	Co-inoculation of all strains	10:10:10:1	1 × 10^7^	1 × 10^7^	1 × 10^7^	1 × 10^6^
10S48	Sequential inoculation of Sc at 48 h	10:10:10:1	1 × 10^7^	1 × 10^7^	1 × 10^7^	1 × 10^6^
10S72	Sequential inoculation of Sc at 72 h	10:10:10:1	1 × 10^7^	1 × 10^7^	1 × 10^7^	1 × 10^6^
10CN	Co-inoculation of non- *Saccharomyces* strains	10:10:10:0	1 × 10^7^	1 × 10^7^	1 × 10^7^	
C	Single inoculation of Sc					1 × 10^6^

**Table 2:** Concentrations of sugars, ethanol, glycerol and organic acids at the end of fermentation.

Compounds	C	1CA	1S48	1S72	1CN	10CA	10S48	10S72	10CN
Residual sugars (g/L)	0.13 ± 0.17	0.39 ± 0.29	1.22 ± 0.62	0.42 ± 0.57	29.61 ± 5.65 *	0.42 ± 0.41	0.37 ± 0.22	0.88 ± 1.03	28.30 ± 6.29 *
Ethanol production (%, *v*/*v*)	13.06 ± 0.03	12.93 ± 0.06	12.58 ± 0.10 *	12.57 ± 0.08 *	11.04 ± 0.13 *	12.77 ± 0.05 *	12.41 ± 0.11 *	12.29 ± 0.06 *	10.91 ± 0.06 *
Ethanol yield (g/g)	0.47 ± 0	0.46 ± 0	0.45 ± 0 *	0.45 ± 0 *	0.46 ± 0.01	0.46 ± 0	0.45 ± 0 *	0.44 ± 0 *	0.45 ± 0.01 *
Ethanol reduction (%, *v*/*v*)	0	0.13 ± 0.06	0.48 ± 0.10 *	0.49 ± 0.08 *	NC	0.29 ± 0.05 *	0.65 ± 0.11 *	0.77 ± 0.06 *	NC
Glycerol (g/L)	6.57 ± 0.08	6.57 ± 0.22	5.41 ± 0.75 *	5.74 ± 0.93	4.96 ± 0.41 *	6.23 ± 0.11	6.70 ± 0.48	6.38 ± 0.18	5.44 ± 0.68 *
Citric acid (g/L)	0.13 ± 0	0.13 ± 0	0.11 ± 0.01 *	0.12 ± 0.01 *	0.12 ± 0 *	0.12 ± 0.01 *	0.12 ± 0.01 *	0.11 ± 0 *	0.11 ± 0 *
Tartaric acid (g/L)	2.79 ± 0.07	3.10 ± 0.14	3.41 ± 0.38	3.30 ± 0.31	3.38 ± 0.34	2.47 ± 1.10	3.23 ± 0.25	3.44 ± 0.14	3.38 ± 0.28
Malic acid (g/L)	0.81 ± 0.13	0.63 ± 0.05 *	0.50 ± 0.03 *	0.45 ± 0.03 *	0.39 ± 0.03 *	0.61 ± 0.02 *	0.54 ± 0.03 *	0.51 ± 0.01 *	0.44 ± 0.02 *
Succinic acid (g/L)	0.44 ± 0	0.43 ± 0.05	0.40 ± 0.05	0.36 ± 0.05	0.37 ± 0.02	0.44 ± 0.06	0.52 ± 0.03	0.52 ± 0.02	0.67 ± 0.05 *
Lactic acid (g/L)	0.20 ± 0.03	0.24 ± 0.02	0.24 ± 0.01	0.30 ± 0.10 *	0.29 ± 0.01 *	0.23 ± 0.05	0.30 ± 0 *	0.33 ± 0.04 *	0.25 ± 0.03
Acetic acid (g/L)	0.10 ± 0.01	0.14 ± 0.03	0.29 ± 0.08 *	0.36 ± 0.05 *	0.32 ± 0.06 *	0.22 ± 0.05 *	0.23 ± 0.05 *	0.22 ± 0.08 *	0.21 ± 0.04 *

Values are mean ± standard deviation of three independent replicates; The initial sugar concentration of synthetic must was 220 g/L; * means statistically significant differences from the control sample of C on the same row (LSD test, *p* < 0.05); NC means no calculate ethanol reduction due to stuck fermentation.

## Data Availability

The data presented in this study are available on request from the corresponding author.

## Appendix A

**Table A1:** Primer sequences used for quantitative PCR analysis.

Target Species	Primer Name	Primer Sequence 5′-3′	References
*S. cerevisiae*	CESP-F	ATCGAATTTTTGAACGCACATTG	Hierro et al. (2007) [28]
	SCER-R	CGCAGAGAAACCTCTCTTTGGA	
*M. pulcherrima*	MP2-F	AGACACTTAACTGGGCCAGC	García et al. (2017a) [38]
	MP2-R	GGGGTGGTGTGGAAGTAAGG	
*T. delbrueckii*	Tods L2	CAAAGTCATCCAAGCCAGC	Zott et al. (2010) [39]
	Tods R2	TTCTCAAACAATCATGTTTGGTAG	
*Z. bailii*	ZBF1	CATGGTGTTTTGCGCC	Rawsthorne and Phister. (2006) [26]
	ZBR1	CGTCCGCCACGAAGTGGTAGA	

**Table A2:** Slopes, Y-Intersections, correlation coefficients (R^2^), efficiencies (%), limits of quantification (LoQ) and limits of detection (LoD) of standard curves obtained from serially diluted DNA of *S. cerevisiae*, *M. pulcherrima*, *T. delbrueckii* and *Z. bailii* with PMAxx treatment. Efficiency was estimated by the formula E = (10^−1/slope^) − 1.

	Slope	Y-Intersection	R^2^	Efficiency (%)	LoQ	LoD
*S. cerevisiae*	−3.45 ± 0.04	40.38 ± 0.20	0.9985	94.80	10^3^	10
*M. pulcherrima*	−3.40 ± 0.03	43.51 ± 0.19	0.9991	97.01	10^4^	10^2^
*T. delbrueckii*	−3.35 ± 0.04	40.02 ± 0.25	0.9978	98.83	10^3^	10
*Z. bailii*	−3.65 ± 0.03	41.81 ± 0.17	0.9986	87.92	10^3^	10

**Table A3:** The concentration of assimilable nitrogen and the added concentration on the 2nd and 3rd day of fermentation.

Days	Fermentation	Nitrogen (mg/L)	Added Nitrogen (mg/L)
2nd day	1S48	102 ± 5.66	0
	10S48	79.67 ± 9.02	20 ± 5.50
3rd day	1S72	70.67 ± 13.05	31 ± 8.54
	10S72	48 ± 5.29	53.33 ± 5.77
